# Open castration in dromedary camel through two scrotal incisions: A case report

**DOI:** 10.1002/ccr3.6931

**Published:** 2023-02-08

**Authors:** Solomon Amente Adugna, Jiregna Dugassa Kitessa

**Affiliations:** ^1^ Department of Veterinary Medicine, School of Veterinary Medicine Wallaga University Nekemte Ethiopia; ^2^ Department of Clinical Studies, College of Veterinary Medicine and Agriculture Addis Ababa University Bishoftu Ethiopia

**Keywords:** case report, castration, dromedary camel, lidocaine hydrochloride, orchitis

## Abstract

Most of the time, castration is an elective procedure performed to make the camel docile and manage its behavior. There are also instances, where it is performed as a treatment of testicular abnormalities such as orchitis, irreparable traumatic injuries, and tumors. In the current study, a 7‐year‐old bull dromedary camel was brought to Addis Ababa University College of Veterinary Medicine and Agriculture, Feseha Gebre Ab VTH, with a history of recurrent swelling of the left testicle for a month. Based on history and clinical examination, the case was diagnosed as unilateral orchitis and admitted for open castration of both testes. Two percent lidocaine hydrochloride was injected into each testicle to provide a spermatic nerve block, and then, two parallel incisions were made through the scrotal skin on either side of the median raphe. The testicles were pulled out through these incisions, ligated, and transected. The scrotal incisions were left open to heal by themselves. Postoperatively, antibiotic and anti‐inflammatory drugs were administered for 3 successive days. Finally, the camel was regularly followed for 1 week and recovers uneventfully.

## Graphical abstract

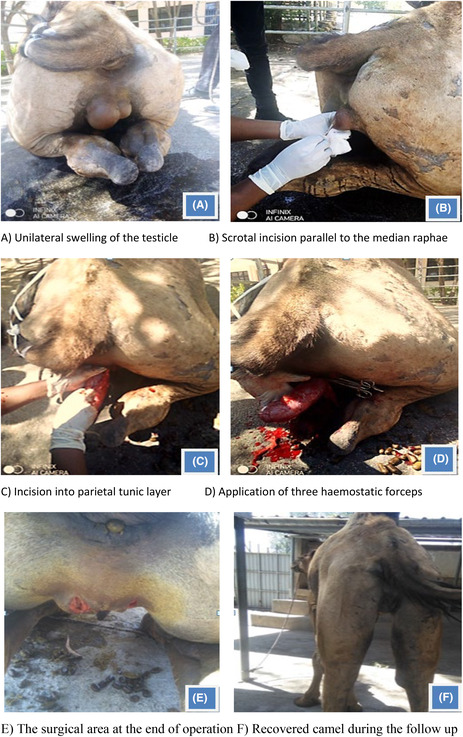

## 1 INTRODUCTION

Most of the time, castration is an elective procedure performed to render the camel docile and manage its behavior because it can make them more manageable and easier to work with.^1^ Castrated animals sometimes make a better captive display animal as they do not come into a rut and are less likely to become aggressive toward keepers or the public. Sometimes, castration is also performed to avoid accidental mating from the inferior sire and in rare conditions such as testicular abnormalities including orchitis, irreparable traumatic injuries, and tumors.^2^ The ideal age to castrate a male camel is between 4 and 6 years old.^3^ Castration is done in camels in a standing position or recumbent position.^1^

The routine castration process involves an incision over the scrotum at both sides and removing the testicle after ligation of the vascular portion of the spermatic cord, and wounds are left open for postoperative care like in equines.^2^ The recently developed prescrotal midline incision method does not require any postoperative care.^1^ Primary bleeding may be a potential complication if the vascular portion of the spermatic cord is not properly ligated or emasculated, and in cases where the surgical procedure is not performed according to the standard surgical principles, infection of the surgery site may take place necessitating meticulous postoperative care. In rare instances, the formation of a unilateral or bilateral scirrhous cord as a result of chronic infection may necessitate an elaborate second surgical procedure.^3^

Unlike in horses, there is negligible postoperative drainage in the camel following open castration. This precluded the need for postoperative care as compared to when the scrotal wounds were left open.^2^ However, in a lately modified technique and instead of two incisions, a single, small prescrotal midline incision was used to exteriorize both the testicles through it and the incision was closed with one interrupted horizontal mattress suture using absorbable suture material of the proper size and assumed to give encouraging outcome and relatively cosmetic, due to small scar in the prescrotal midline region.^1^ This case report aimed to describe the surgical sterilization of the dromedary camel by open castration through two parallel scrotal incisions using a spermatic nerve block.

## 2 DESCRIPTION OF THE CASE

### 2.1 Case history and clinical examination

**FIGURE 1:**
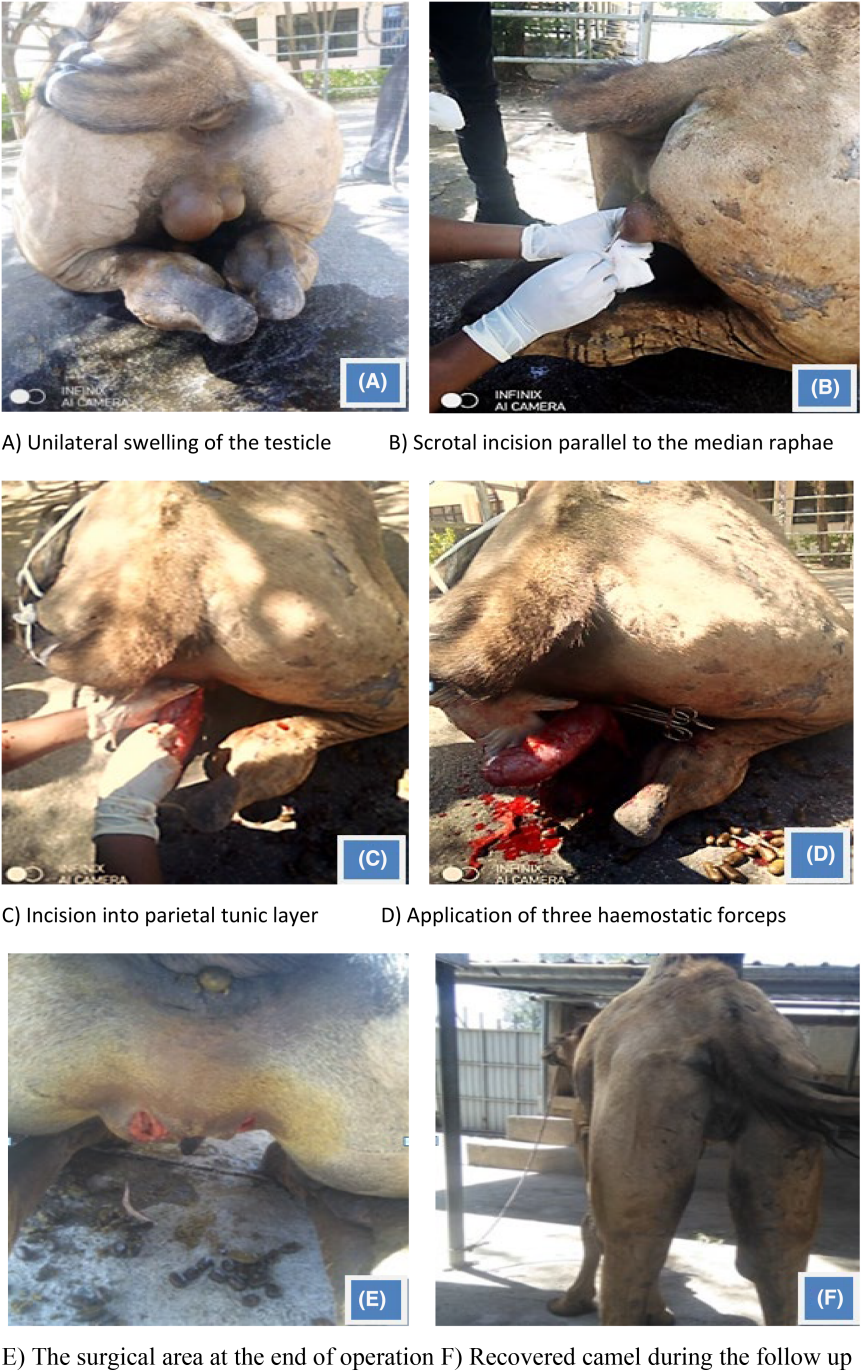
Progression of open castration in dromedary camel.

A 7‐year‐old bull dromedary camel weighing 400 kg was presented to Addis Ababa University College of Veterinary Medicine and Agriculture, Feseha Gebre Ab Veterinary Teaching Hospital, with a history of recurrent swelling of the left testicle for a month. The problem was treated medically using PenStrep, 1 mL/20 kg, IM, and SID for 5 days before 3 weeks, and the swelling regressed for a while but re‐occurred again and then referred for surgical treatment. Upon clinical examination and palpation of the testicle, the left testicle was enlarged, hot, and painful to touch (Figure 1A). Based on history and systemic physical examination, the case was diagnosed as unilateral orchitis and admitted for open castration of both testicles using local anesthesia.

### 2.2 Preoperative preparation

The camel was controlled in sternal recumbence with physical restraint, the tail was tied to the side using gauze, and the scrotal area was prepared aseptically by washing, shaving, and scrubbing with diluted tincture iodine. Appropriate precautions were taken into account to prevent the spread of zoonotic diseases like Brucellosis to the surgeon.

### 2.3 Anesthesia and animal control

The animal was restrained in sternal recumbence using a rope tied to its legs by passing over its neck and hump. Then, 2% lidocaine hydrochloride @ 0.5 mg/kg was injected into each testicle for spermatic nerve block.

### 2.4 Surgical procedure and technique

The right testicle was grasped caudoventrally between the thumb and index finger of the left hand, and a 4 cm vertical incision was made over the mid‐part of the scrotum (Figure 1B). The testicle was exteriorized through the incision line, and then, the parietal vaginal tunic was incised to allow direct ligation of the spermatic cord (Figure 1C). Three hemostatic forceps were placed on the spermatic cord, and the cord was double ligated by circumferential transfixation ligatures using absorbable suture material Polyglycolic acid size # 2 (Figure 1D). Then, the testicle was removed by severing the cord between the middle forceps and the one closest to the testicle. The opposite testicle was exteriorized from the other parallel incision and removed in the same manner. After the removal of both testicles, procaine penicillin powder was applied to the surgical wounds and the surgical wound was left open to heal by second intention (Figure 1E).

### 2.5 Postoperative care and outcome

Postoperatively, broad‐spectrum antibiotic procaine penicillin (24 mg/kg) and dihydrostreptomycin sulfate (30 mg/kg) (Pen &Strep® Norbook UK), @1 mL/20 kg, IM, SID, and anti‐inflammatory drug dexamethasone sodium phosphate injection, 0.1 mg/kg, 10 mL, IM, SID, were administered for 3 successive days. The surgical wound was dressed with antiseptics every other day for 3 days. Finally, the camel recovered uneventfully after 1 week of regular follow‐up (Figure 1F).

## 3 DISCUSSION

Castration of male camels is one of the routine management surgical practices throughout the world. It is mostly performed to reduce their aggressive behavior, especially during the rutting season, and testicular diseases.^3^ Similarly in the present case management, it was performed for the treatment of recurrent unilateral orchitis. In most males, castration is recommended at 2 years of age; however, earlier castration (6 months) is also possible.^1^ But in the current case report, the camel was aged 7 years. Several types of anesthetic regimes can be used including chloroform, etorphine with acepromazine, chloral hydrate, and xylazine.

In field conditions, the best results for security (and lower cost) are sedation with xylazine and local anesthesia.^2^ However, in the case at hand, the camel was restrained physically in a sitting position and local anesthesia of 2% lidocaine hydrochloride was infiltrated intratesticular. This case management is also similar to the technique used by the authors,^4^ the camel was controlled properly, and the scrotum is injected with 2% lidocaine solution 3 to 5 cm cranial to the scrotum on either side of the median raphe. Castration using the Burdizzo method has been used in the past but is now abandoned in favor of the complete removal of the testis. So, the technique used nowadays is similar to standard methods used in other species.^5^

In the present case, two skin incisions were made parallel to the median raphe through which the testicles were exteriorized, transected, and the scrotal incisions were left open to allow drainage. A similar surgical technique was reported by the authors.^2^ Different approaches are used in camel castration. Of these, a prescrotal castration technique is preferred due to its aesthetic and limited surgical scar.^1^ However, in the current case, the prescrotal technique was not applied due to the absence of deep sedation. Generally, in the present case report, open castration using two scrotal incision techniques was successfully conducted in the dromedary camel without any complications.

## 4 CONCLUSION AND RECOMMENDATIONS

Castration is an elective procedure that is normally done to render the camel docile and easily controllable. Sometimes, castration is done to avoid accidental mating when the animal is not desirable for breeding purposes and in certain testicular abnormalities such as orchitis, irreparable traumatic injuries, and tumors The standard surgical procedure involves open castration in which both scrotal sacs and the tunica vaginalis cavities are opened through two large incisions, the testes removed, and the wounds left open for postoperative care until complete healing takes place.

Based on the above conclusion, the following points were recommended:

- The lately modified technique and instead of two incisions, a single, small prescrotal midline incision should be made to exteriorize both the testicles through it, and the incision should be closed with one interrupted horizontal mattress suture.
- The procedure should be performed by observing the general surgical principles to minimize the complication of postoperative sepsis.

## AUTHOR CONTRIBUTIONS

**Solomon Amente Adugna:** Conceptualization; data curation; investigation; methodology; validation; writing – original draft. **Jiregna Dugassa Kitessa:** Validation; writing – review and editing.

## FUNDING INFORMATION

No fund was provided for this case report.

## CONFLICT OF INTEREST STATEMENT

The authors declare no conflict of interest.

## ETHICS STATEMENT

The authors confirm that the ethical policies of the journal, as noted on the journal's author guidelines page, have been adhered to and the appropriate ethical review committee approval has been received.

## CONSENT

Written informed consent was obtained from the patient to publish this report in accordance with the journal's patient consent policy.

## Data Availability

Since this is a case report no data was recorded.

## References

[1] Telfah MN , Siddiqui MI , Taleb SA . Castration of dromedary camel through prescrotal midline incision. Open Vet J. 2012;2(1):106‐108. 26623301 PMC4655769

[2] Chittora RK , Upreti NC , Yadav CD , Jadhav AS . Castration of camels through prescrotal midline incision. J Camel Pract Res. 2020;27(1):139‐140.

[3] Moretti J . Husbandry guidelines for Arabian camel *Camelus dromedarius* , 2008, 152.

[4] Lapidge SJ , Eason CT , Humphrys ST . A review of chemical, biological and fertility control options for the camel in Australia. Rangel J. 2010;32(1):95‐115.

[5] Larson J . Information resources on the south American camelids: llamas, alpacas, guanacos, and vicunas, 1967–2003, 2007.

